# Ultra-Processed Food Consumption during Pregnancy and Its Association with Maternal Oxidative Stress Markers

**DOI:** 10.3390/antiox11071415

**Published:** 2022-07-21

**Authors:** Ameyalli M. Rodríguez-Cano, Isabel González-Ludlow, Blanca V. Suárez-Rico, Araceli Montoya-Estrada, Omar Piña-Ramírez, Sandra B. Parra-Hernández, Enrique Reyes-Muñoz, Guadalupe Estrada-Gutierrez, Claudia C. Calzada-Mendoza, Otilia Perichart-Perera

**Affiliations:** 1 Section for Postgraduate Studies and Research, Higher School of Medicine, Instituto Politécnico Nacional, Plan de San Luis y Díaz Mirón s/n, Casco de Santo Tomas, Miguel Hidalgo, Mexico City 11340, Mexico; rocameyalli@gmail.com (A.M.R.-C.); ccalzada@ipn.mx (C.C.C.-M.); 2 Nutrition and Bioprogramming Coordination, Instituto Nacional de Perinatología Isidro Espinosa de los Reyes, Montes Urales 800, Lomas de Virreyes, Miguel Hidalgo, Mexico City 11000, Mexico; isaglezludlow@icloud.com; 3 Community Interventions Research Branch, Instituto Nacional de Perinatología Isidro Espinosa de los Reyes, Montes Urales 800, Lomas de Virreyes, Miguel Hidalgo, Mexico City 11000, Mexico; blancasuarezrico@gmail.com; 4 Gynecological and Perinatal Endocrinology Coordination, Instituto Nacional de Perinatología Isidro Espinosa de los Reyes, Montes Urales 800, Lomas de Virreyes, Miguel Hidalgo, Mexico City 11000, Mexico; ara_mones@hotmail.com (A.M.-E.); dr.enriquereyes@gmail.com (E.R.-M.); 5 Bioinformatics and Statistical Analysis Department, Instituto Nacional de Perinatología Isidro Espinosa de los Reyes, Montes Urales 800, Lomas de Virreyes, Miguel Hidalgo, Mexico City 11000, Mexico; delozath@gmail.com; 6 Immunobiochemistry Department, Instituto Nacional de Perinatología Isidro Espinosa de los Reyes, Montes Urales 800, Lomas de Virreyes, Miguel Hidalgo, Mexico City 11000, Mexico; rebe1602@hotmail.com; 7 Research Division, Instituto Nacional de Perinatología Isidro Espinosa de los Reyes, Montes Urales 800, Lomas de Virreyes, Miguel Hidalgo, Mexico City 11000, Mexico; gpestrad@gmail.com

**Keywords:** diet quality, fiber, malondialdehyde, protein carbonylation, total antioxidant capacity

## Abstract

Ultra-processed food (UPF) consumption during gestation may lead to increased oxidative stress (OS) and could affect pregnancy outcomes. This study aims to evaluate the association of UPF consumption during pregnancy with circulating levels of OS markers. Diet was assessed (average of three assessments) in 119 pregnant women enrolled in the OBESO perinatal cohort (Mexico), obtaining quantitative data and the percentage of energy that UPFs (NOVA) contributed to the total diet. Sociodemographic, clinical (pregestational body-mass index and gestational weight gain) and lifestyle data were collected. Maternal circulating levels of OS markers (malondialdehyde (MDA), protein carbonylation (PC), and total antioxidant capacity (TAC)) were determined at the third trimester of pregnancy. Adjusted linear regression models were performed to analyze the association between UPFs and OS markers. UPFs represented 27.99% of the total energy intake. Women with a lower UPF consumption (<75 percentile°) presented a higher intake of fiber, ω-3, ω-6, and a lower ω-6/3 ratio. Linear regression models showed that UPFs were inversely associated with TAC and MDA. Fiber intake was associated with PC. UPF intake during pregnancy may result in an increase in oxidative stress. When providing nutrition care, limiting or avoiding UPFs may be an intervention strategy that could promote a better antioxidant capacity in the body.

## 1. Introduction

Fetal development is now recognized as a critical period in the etiology of human disease. The concept of fetal programming suggests that maternal nutritional imbalances and metabolic disorders have a persistent and intergenerational effect on offspring health and disease risk, including obesity, cardiovascular disease (CVD), type 2 diabetes (T2DM), and cancer [1,2]. Deviations in the quality or quantity of nutrients consumed by the mother can exert metabolic changes in the intrauterine environment, leading to the reprogramming of fetal tissues [3,4].

Different maternal situations, including malnutrition and excessive energy and nutrient intake, have been associated with oxidative stress (OS) [3,4], which not only has an impact on maternal health, but may compromise fetal programming [4]. Approximately 5% of the oxygen used in the body is converted into free radicals, highly reactive molecules with unpaired electrons, also known as reactive oxygen species (ROS). Mitochondria are the main source of ROS, as a by-product of ATP production through electron leakage from the respiratory chain [5,6]. The physiological levels of ROS play an important regulatory role through various signaling transduction pathways in folliculogenesis, oocyte maturation, corpus luteum and uterine function, embryogenesis, embryo implantation, and fetoplacental development [7]. However, in a situation of chronic ROS production, the neutralizing capacity is exceeded, causing OS, which is a disturbance in the redox balance of the cell, and resulting in the excessive oxidation of intracellular compounds [5], inducing cell damage and an inflammatory environment [6]. The normal response of the cell to the production of ROS is mediated through the antioxidant system [5,8,9], which ensures an adequate defense against OS. Nutritional deficiencies of proteins and/or micronutrients can affect the capacity of cellular antioxidants [7].

Increased OS levels have been linked to several perinatal complications, including gestational diabetes mellitus (GDM), miscarriage, idiopathic recurrent pregnancy loss, defective embryogenesis, preeclampsia (PE), intrauterine growth restriction, and preterm [10,11].

The Western dietary pattern, associated with a higher risk of metabolic disorders, is characterized by a high consumption of ultra-processed foods (UPF) [6,12], which are high in energy, total as well as saturated and trans fats, and added sugars [13], and because of their hyperpalatable nature, they promote overconsumption. A high intake of UPFs may lead to an increased metabolic load of the mitochondria, resulting in an active respiratory chain that can form excessive ROS [6,14,15]. The consumption of UPFs, according to the NOVA classification, has been proposed as a predictive indicator of the quality of the diet, reflecting a diet with a high-energy density, high added sugars, and high fat, but with low fiber and micronutrient content [16,17,18,19]. This system classifies foods according to the nature, extent, and purpose of the industrial processing through which the food is subjected [16,20]. UPF consumption has increased worldwide, affecting all age groups [21], representing 25% and up to 80% of the total daily energy intake [22,23,24,25,26]. Its consumption has been positively associated with obesity [23,27], abdominal obesity [26], hypertension [28,29], and cancer [22]. An ultra-processed- food-based diet has shown to be associated with a higher energy intake, higher meal eating rate, lower satiety, and higher appetite hormones [30]. To date, it is not clear how UPFs could affect pregnancy. The aim of this study is to investigate the association between the consumption of UPFs during pregnancy and markers of OS in a group of Mexican women.

## 2. Materials and Methods

### 2.1. Setting and Study Population

This is a secondary analysis from the OBESO (Origen Bioquímico y Epigenético del Sobrepeso y la Obesidad) perinatal cohort (2017–2020), which aimed to define the different determinants of obesity programming and was conducted at the Instituto Nacional de Perinatología Isidro Espinosa de los Reyes, in Mexico City, Mexico. The cohort characteristics have been described previously [31]. Women who agreed to participate signed an informed consent.

The OBESO cohort was carried out in accordance with the Declaration of Helsinki and was approved by the Institutional Committees of Ethics and Research (Register number: 3300-11402-01-575-17). Women were recruited by convenience, including adult women (≥18 yo) with a single pregnancy, with a pregestational body mass index (BMI) ≥18.5, and without previous diseases (T2DM, high blood pressure, uncontrolled thyroid disorders, heart disease, autoimmune, kidney, liver diseases, HIV, and HPV). The following were excluded: women with medications that alter inflammation and/or OS markers of chronic use (steroids, non-steroidal anti-inflammatory drugs such as ibuprofen and naproxen, antiretrovirals, antineoplastics, among others), with tobacco, drug, or alcohol consumption, or with finding of congenital structural malformations in the fetus or abnormal fetal karyotype. We eliminated from this analysis women who did not have complete diet information (3 evaluations) or without a blood sample in the 3rd trimester. Women were recruited at the Maternal-Fetal Medicine Department in the first trimester (<14 weeks) of pregnancy. Women had a total of 3 follow-ups, with one visit each trimester of pregnancy. In the first trimester, sociodemographic and clinical information was collected, including age (years), educational level (basic: elementary school and/or incomplete middle school; middle: completed middle school or high school; and higher: technical career, bachelor’s degree and/or graduate degree), occupation (student/employee and housewife), socioeconomic status (very low, low, and middle/high), parity (nulliparous—no previous pregnancy or multiparous—≥1 previous pregnancy). Women reported their pregestational weight, height was measured (according to Lohman’s technique [32]) to the nearest 0.1 cm using a digital fixed stadiometer (model 264, SECA, Hamburg, Germany) and pregestational BMI was obtained, using the WHO criteria for classification [33]. In each visit, the gestational weight gain (GWG) was determined according to the recommendations established by the IOM [34] and calculated with the measured current weight (with light clothing, without shoes; measured to the nearest ±0.1 kg with a calibrated digital scale; BMB-800, TANITA, Tokyo, Japan). Women were asked about their consumption of multivitamin supplements, and the total number of trimesters of multivitamin use was recorded. Physical activity was evaluated through the Pregnancy Physical Activity Questionnaire (PPAQ) [35], obtaining MET-hours/week (metabolic equivalent of task-hours/week). In the 1st and 3rd trimesters, sleep quality was assessed with the Pittsburgh scale (score from 1 to 21, higher scores represent worst sleep quality) [36,37]. The presence of GDM, PE, and/or preterm birth was obtained from institutional clinical records.

### 2.2. Dietary Assessment

The diet was assessed in each trimester using a multiple pass 24 h (24 h) recall (for a total of 3 evaluations), applied by a trained nutritionist, using food replicas and standard measuring cups, spoons, and glasses. Nutritional analysis was obtained through the Food Processor SQL software (version 14.0, ESHA Research, Salem, OR, USA), which included standardized recipes and Mexican foods (using Mexican Tables of Nutritional Value or food labels) in the database. The consumption of energy (kcal), macronutrients (as grams (g) and as a percentage of total energy intake (%TEI)), fiber (g), monounsaturated fatty acids (MUFA) (g, %TEI), polyunsaturated fatty acids (PUFA) (g, %TEI), PUFA ω-3 (g, %TEI), PUFA ω-6 (g, %TEI), saturated fatty acids (SFA) (g, %TEI), and trans fatty acids (TFA) (g, %TEI) was obtained. The ratio PUFA ω-6/3 was calculated. To establish the usual intake, the average of the 3 dietary assessments was computed.

### 2.3. Ultra-Processed Food Consumption

The categorization of UPFs was conducted according to the NOVA definition [16,38]. From the “detail” section of the 24 h recall, UPFs were identified: nutrition labels of each one of the products were analyzed. In the case of missing or uncertain information, e.g., because the patient did not know the brand or origin, the most common type of food consumed in our population was standardized and used. From the nutritional analysis, the energy of each UPF identified was obtained, as the percentage of its contribution to the total energy value of the diet. The sum of all the UPFs from one day was computed (%UPF of the diet). The average of %UPF from the 3 dietary assessments was obtained. According to the 75th percentile (75°) of UPF intake, women were classified as having a “lower intake” or “higher intake”, when consumption was <75° or ≥75°, respectively.

### 2.4. Oxidative Stress Markers

At the 3rd trimester visit, a fasting blood sample was obtained to determine markers of OS. Blood samples were collected in K2EDTA Vacutainer tubes (Becton-Dickinson, Franklin Lakes, NJ, USA) and centrifuged for 15 min at 1000× *g*. Serum samples were stored at −70 °C until the assays were performed. Malondialdehyde (MDA), the end-product of lipoperoxidation, is the most representative marker of oxidative lipid damage. MDA was determined in plasma, according to Gérard-Monnier et al. [39], adding 1-methyl-2-phenylindole (MPI) and 37% HCl. The reaction was incubated at 45 °C for 40 min. After the incubation time, the samples were centrifuged at 10,000 rpm for 5 min; absorbance was determined at 584 nm. To perform the type curve, tetraethoxypropane (TEP) was used as a standard solution. The values obtained are expressed as nmol of MDA/mg dry weight. Protein carbonylation (PC) was determined by the dinitrophenylhydrazine method [40]. Briefly, 50 µL of plasma was mixed with 0.5 mL 10 mM dinitropenylhydrazine (DNPH) in 2.5 M HCl (or 2.5 M HCl alone for the blank). Samples were left for 1 h at ambient temperature, and then 0.5 mL 20% trichloroacetic was added and centrifugated at 3000 rpm, 5 min, 4 °C. The resultant pellet was rinsed twice by centrifugation with 1mL 5% TCA. The pellet was washed by centrifugation with 2 mL ethanol/ethylacetate (1:1) and solubilized in 0.5 mL of 6M guanidine in 20 mM potassium phosphate, pH 2.3. The carbonyl concentration was determined using the extinction molar coefficient e = 22 mM^−1^ cm^−1^ and expressed as nmol/mg protein. Total antioxidant capacity (TAC) as indicative of an antioxidant defense system in plasma was evaluated according to a method based on the cupric-reducing antioxidant capacity (CUPRAC), using copper (II) and neocuproine reagents. The absorbance was determined at 450 nm. The results were expressed as pmol Trolox equivalent/mg protein. Trolox is a water-soluble analog of vitamin E [41]. The OS markers presented in this analysis have high sensitivity and reproducibility and use validated methods.

### 2.5. Statistical Analysis

Descriptive statistical tests (mean and standard deviation, frequencies, median and interquartile range) were performed to present the characteristics of the population, as well as the description of the diet and OS markers. To evaluate the association between the consumption of UPFs and biochemical markers, bivariate analyses were performed through correlations (Pearson/Spearman), mean differences (Student’s *t*-test/Mann–Whitney U-test and one-way ANOVA/Kruskal–Wallis), and differences between proportions (chi-squared test). SPSS software version 26.0 (IBM, Armonk, NY, USA) was used.

Different linear regression models were performed to analyze the association between the consumption of UPFs and each of the OS markers, adjusted for dietary, sociodemographic (age, educational and socioeconomic level, and occupation), clinical (pregestational BMI, GWG, parity, and multivitamin consumption), perinatal (complications), and lifestyle (physical activity and sleep quality) variables. The Recursive Feature Elimination technique was performed to select the relevant variables included in the models. This method computes several Theil–Sen regressions iteratively using different subsets of variables each time and removing those that do not contributed to the phenomena description. In the case that pregestational BMI was not selected, it was manually added. After the feature selection, for each target output, their optimal regression model was selected using the minimal Akaike information criterion. The final models consisted of ordinary least squared regression considering linear-robust M-Estimation and a robust covariance estimation using the HC3 case. These analyses were performed using the libraries scikit-learn and statsmodels from Python 3.9. To consider significant findings, a value of *p* < 0.05 was considered.

## 3. Results

Of a total of 201 patients included in the cohort, 25 women were excluded because they used medications (*n* = 8), had chronic non-communicable diseases before pregnancy (T2DM, hypertension, and uncontrolled thyroid disease) (*n* = 14), or pregnancy complications (cholestasis, *n* = 3). Fifty-seven women were eliminated due to lack of follow-up or incomplete diet data. The final sample included a total of 119 pregnant women.

### 3.1. Participants’ Characteristics

Mean age of women was 29.52 ± 4.97 years; 53.6% (*n* = 60) of them were nulliparous and 67.6% (*n* = 71) were housewives. More than a half of women were classified as very low/low socioeconomic status (62.4%, *n* = 74). As for educational level, 20.2% (*n* = 24) of women reported basic and 42.0% (*n* = 50) higher level.

Pregestational BMI mean value was 26.52 ± 4.69 kg/m^2^; 42% (*n* = 50) of women were classified as having a normal weight, while 36.1% (*n* = 43) and 21.8% (*n* = 26) were overweight and obese, respectively. On average, physical activity during pregnancy was 203.76 ± 92.30 MET-hours/week, while the average sleep quality score was 8.44 ± 2.40. Regarding the consumption of supplements, 58.8% (*n* = 70) of the women consumed multivitamins (which included iron and folic acid) during the three trimesters; only 5.9% (*n* = 7) of them never used multivitamins during pregnancy. In the third trimester visit, 43.7% (*n* = 52) presented excessive GWG, 31.1% (*n* = 37) insufficient, and only 25.2% (*n* = 30) adequate GWG. The resolution of pregnancy was on average at 38.81 ± 1.52 weeks of gestation (according to first trimester US). GDM/PE was present in 13.6% (*n* = 16) of women and premature births represented 8.8% (*n* = 10).

### 3.2. Ultra-Processed Food Consumption during Pregnancy

The consumption of UPFs is described in Table 1; the minimum and maximum consumption found were 3.44% and 57.69%, respectively. Lower or higher UPF intake was considered if consumption was <34.62% or ≥34.62% (corresponding to the 75°). No differences were found in the consumption of UPFs according to maternal sociodemographic and clinical variables (Table 1). The consumption of UPFs presented a negative correlation with pregestational BMI (*r*= −0.218; *p* = 0.017) and a positive correlation with physical activity (*r* = 0.196; *p* = 0.033).

Mean consumption of energy and macronutrients during pregnancy and according to UPF intake are shown in Table 2. Women classified <75° of UPF consumption presented a higher intake of fiber, PUFA ω-3 (g and %TEI), and PUFA ω-6 (%TEI) and a lower ω-6/3 ratio (Table 2).

Compared to women with normal pregestational BMI, those with obesity had a lower intake of energy (kcal: normal, 2226.67 ± 628.73; overweight, 2044.79 ± 458.57; Obesity, 1914.79 ± 445.82; *p* = 0.045) and of carbohydrates (g: normal, 291.07 ± 80.46; overweight, 259.40 ± 64.09; obesity; 248.63 ± 60.02; *p* = 0.038). No other differences in diet were found by pregestational BMI.

### 3.3. Oxidative Stress Markers in Pregnancy

The concentrations of OS markers at the end of pregnancy are described in Table 3. Women with pregestational obesity had a higher concentration of MDA (*p* = 0.002) and PC (*p* < 0.001), and pregestational BMI presented a positive correlation with MDA (*r* = 0.272, *p* = 0.003) and with PC (*r* = 0.432, *p* < 0.001). There were no differences in OS markers according to socioeconomic status, educational level, occupation, parity, third trimester GWG, the presence of complications, or according to the use of multivitamins (Table 3). Age presented positive correlation with TAC (*r* = 0.286; *p* = 0.002). Physical activity and sleep score were not correlated with biochemical markers.

### 3.4. Association of UPF Consumption and OS Markers

UPF presented a negative correlation with TAC (*r* = −0.224; *p* = 0.015). A higher TAC was found in those <75° of UPF intake (*p* = 0.041) (Table 3). There were no differences in the concentration of MDA or PC according to the percentile of UPF consumption (Table 3).

The linear regression models showed that UPF was negatively associated with MDA (*p* < 0.001). In the case of the PC model, pregestational BMI and fiber showed a positive association with PC (*p* = 0.045), where UPF did not show relevance for PC. As for the TAC model, UPFs were negatively associated and MVI use was positively associated with TAC (*p* < 0.001) (Table 4).

## 4. Discussion

This study presents data on some aspects of diet quality during pregnancy, which are scarce in the literature. In this study, UPF consumption during pregnancy represented around 30% of the total energy intake (TEI); a higher UPF intake was associated with a lower TAC and MDA. Other elements of the diet, such as fiber, were associated with PC.

UPF consumption is associated with diet quality, indicating an adverse nutritional profile. Our results show that a lower UPF consumption was related to a higher intake of fiber, PUFA ω-3, and PUFA ω-6 and a lower ω-6/3 ratio. In Mexico, UPF sales increased 29.2% (from 164.3 to 212.2 kg per capita/year) between 2000 and 2013, placing our country in the first place in UPF consumption in Latin America and fourth worldwide. In a recent analysis of 15 national surveys, the energy contribution of UPFs to households; purchases increased from 10.5% to 23.1% kcal from 1984 to 2016 [42]. The national consumption of UPFs in Mexico (excluding pregnant women) represented 30% of TEI, with a contribution in the first quintile of 4.5% and up to 64.2% kcal in the highest quintile [43]. Our results are in line with the national report and are within reported data from other countries: Brazil reported a consumption of 21.5% [13], United States 57.5% [26], Canada 47.7% [25], and France 18.7% [22]. Recently, Sartorelli et al. [44] reported a 32% of UPFs in the diet of Brazilian pregnant women, which is also similar to the result in our sample.

UPF consumption was inversely associated with TAC. UPFs are energy-dense and high in total and saturated fats and added sugars [45]. These different macronutrients have been associated with an increase in the production of mitochondrial ROS and a gradual decrease in the production of ATP. The exacerbated production of ROS leads to the oxidation of macromolecules and cell damage, which leads to cell death and the accumulation of cellular debris. This promotes leukocyte infiltration and the activation of proinflammatory mechanisms, increasing damage to surrounding cells, leading to a vicious cycle of OS inflammation. The systemic dissemination of pro-oxidants and pro-inflammatory agents through the blood favors their interaction with other tissues, conditioning a microenvironment conducive to a chronic state of oxidation and inflammation [46]. On the other hand, pregnancy promotes a scenario of increased OS [47]. A decreased activity of the antioxidant system is observed, affecting the ability to counteract the excessive production of ROS, leading to OS damage. OS has been suggested as a promoter of pregnancy complications, including spontaneous abortions, embryopathies, preterm birth, and intrauterine growth restriction associated with PE [8]. Consuming UPFs displaces natural, fresh, and local foods, which leads to a lower content of different micronutrients, such as vitamins A, C, D, E, and B12, niacin, thiamin, riboflavin, pyridoxine, calcium, magnesium, copper, iron, phosphorus, zinc, and selenium. Additionally, UPFs are low in protein and fiber [45]. Many of these nutrients are part of the antioxidant system, as enzymes (superoxide dismutase (SOD), catalase, and glutathione (GSH)), non-enzymatic elements (vitamins C and E, carotenoids, and selenium), or cofactors (zinc in metallothionein, copper in ceruloplasmin, copper –selenium–zinc in SOD, and selenium in GSH peroxidase) [48]. Accordingly, in one model, multivitamin use showed a positive association with TAC concentration. Multivitamin intake could provide nutrients to support the antioxidant system.

Other authors have documented the relationship between dietary intake in pregnancy and the antioxidant capacity of the organism. The consumption of a DASH diet, a plant-based food pattern and low in processed food, during four weeks in pregnant women with GDM, produced increased concentrations of TAC and glutathione, when compared to women with a control diet, who had significant reductions in the mentioned markers [49]. A small study of 12 pregnant women that reported a low consumption of legumes, vegetables, and fruits observed that women’s values of the oxygen radical absorption capacity (ORAC) decreased during pregnancy [50]. A recent Cochrane review (19 clinical trials) found that multiple micronutrient supplementation during gestation reduced the risk of low-birth weight (high-quality evidence) and small-for-gestational age (moderate-quality evidence), without observing differences in perinatal or neonatal mortality (high-quality evidence) when compared with iron/iron+folic acid supplementation [51]. Other reviews have shown similar results with the use of multivitamin during pregnancy [52]. Clinical guidelines emphasize consuming a minimally processed diet, rich in plant-based foods during pregnancy, which provides enough antioxidants to achieve and maintain a satisfactory antioxidant capacity throughout pregnancy that could counteract the negative effects of OS. Among the main elements in obstetric care that lead to a healthy pregnancy are the consumption of a wide variety of foods and adequate and timely individualized vitamin/mineral supplementation [53].

TAC offers an indirect assessment of OS [54]; one of the main advantages of using TAC is that this measurement can be a preliminary indication of a pathological condition [55]. In terms of perinatal health, antioxidant capacity has been a relevant marker associated with various perinatal complications. Women with GDM have shown diminished TAC levels [56,57]. Furthermore, TAC was found to be predictive for GDM (OR = 12.769; CI95%: 2.464–66.182; *p* = 0.002) and a lower TAC was an independent predictor of the need for insulin in GDM patients (OR = 99.471; CI95%: 2.865–3453.061; *p* = 0.011) [58]. Stepan et al. explained that pregnant women with a pathological uterine perfusion showed a lower plasma antioxidant capacity compared to those with normal uterine perfusion (227.3 ± 4.0 vs. 275.2 ± 10.5 mmol/L; *p* < 0.05) and documented a negative correlation between antioxidant capacity and mean pulsatility index of the uterine arteries (*r* = −0.363; *p* < 0.05) [59]. In women with different types of hypertensive disorders of pregnancy (gestational hypertension, chronic hypertension, preeclampsia, and eclampsia), antioxidant markers (antioxidant activity, albumin, and total thiols) were decreased in all hypertensive groups compared to healthy pregnant women. Women with eclampsia, followed by those with pre-eclampsia, had the lowest values. Additionally, OS markers (ischemia-modified albumin and advanced oxidation protein products) were negatively associated with antioxidant activity in every type of hypertensive disorder, suggesting that OS decreased antioxidant status, due to their usage to counteract the excess ROS generated under these conditions [60]. The total antioxidant status was significantly lower in the early onset severe PE group than in the uncomplicated pregnancies one [61]. A recent review states that there is evidence to support the hypothesis that increased OS and a deficient antioxidant defense is involved in the etiology of recurrent pregnancy loss [62].

Contrary to what we expected, the consumption of UPFs was found to be inversely associated with MDA. This could be the result of a confounding effect given by pregestational BMI, where our results show that those with obesity presented a higher concentration of MDA and a lower consumption of total energy and carbohydrates compared to normal weight women. This is a common problem in diet assessment; in fact, under-reporting is more common in people with higher BMI, especially those with obesity, as well as in women with other characteristics such as dissatisfaction with body image, low educational level, and older age [63,64,65,66]. Under-reporters inform lower overall energy and energy intake from fats and carbohydrates [63,64,65]. The foods that are most frequently under-reported are unhealthy foods, such as cakes and pies, salty snacks, cheese, white potatoes, processed meat, soft drinks, spreads, and condiments, which also correspond to UPFs [63,64]. In fact, UPF consumption was negatively correlated with pregestational BMI. Some studies document that, after excluding under-reporters, the associations between diet and some clinical outcomes (e.g., BMI) changed in magnitude or direction, becoming more consistent with hypotheses linking food to obesity as well as making them more reliable [63,64]. Currently, there is no consensus on how to deal with under-reporting in dietary assessment, but special consideration should be taken in populations where there is a high proportion of people prone to this phenomenon (high prevalence of obesity) [63].

Fiber has usually been associated with beneficial outcomes in pregnancy. The studies by López-Yáñez [67] and Kim [68] found that pregnant women with MDA concentrations in the highest tertiles had a lower consumption of high-fiber foods, such as fruits and vegetables, than those in the lowest tertile. The Australian Longitudinal Study for Women’s Health showed that women in the highest quartile of pre-pregnancy fiber intake had a 33% lower risk of GDM compared to women in the lowest quartile. When analyzed by food group, foods with a higher fiber content, such as vegetables, fruits, bread, were associated with a lower risk of GDM [69] and small for gestational age newborns [70], while the intake of white bread and industrial bakery products and pastries was associated with an increased risk of both outcomes [69,70]. Carbohydrate intake and a glycemic load above the median (vs. lower values) increased the risk of pregnancy-induced hypertension [71]. In our analysis, fiber intake positively influenced PC concentration, an unexpected and inconsistent result. One of the possible explanations is that UPFs could be added with fiber (to make them healthier), and concomitantly be high in added sugars or total, saturated, or trans fats, acting as a confounding factor. A study in adults showed that a higher sugar intake was an independent determinant of higher plasma MDA in a multi-adjusted logistic regression model, suggesting that sugar is directly involved in the generation of OS [72]. This hypothesis is of interest and could be further explored, because UPFs not only include foods easily linked to an unhealthy diet, such as cookies, pastries, and snacks, but also many foods that are marketed and perceived as healthy, such as yogurts, snack bars, functional foods, low calorie/fat/carbs products, high-fiber breakfast cereals, or products fortified with nutrients or bioactive components. The generated evidence should guide not only intake recommendations, but also regulation policies related to food marketing and advertising.

Women with pregestational obesity in our sample had higher concentrations of MDA and PC. Our models were adjusted by pregestational BMI, and it showed positive influence in PC concentration. It is well documented that pregnant women with obesity have increased OS [73,74]. Maternal adipose tissue, which is excessive in obesity, is an important source of OS [75]. Obesity is one of the most common concerns in obstetrics [76], with major adverse perinatal outcomes, such as miscarriage, congenital anomalies, and metabolic dysregulation manifesting as PE or GDM, increasing the risk of metabolic programming in utero [77,78]. Obesity is associated with other risk factors, such as poor nutrition, but low-quality diets probably represent an independent risk factor during pregnancy, rather than being mediators of the changes associated with maternal obesity [78].

This study has some limitations. High intra- and interpersonal variability of diet is inherent in dietary assessment and bias could be a problem [79]. Likewise, because metabolism during pregnancy changes constantly (and gastrointestinal symptoms increase), diet may be subject to greater variation [80]. Additionally, under-reporting may have confounded some of our results, with a probable higher consumption in women with obesity. Regarding UPF classification, nutrient information in food labels in Mexico is unclear, incomplete, or non-existent (especially street foods and bulk unpackages products), limiting proper categorization. Sometimes insufficient details were provided regarding a food product, due to an error in the collection method or because the participants did not know such information. Another limitation is that we only analyzed OS markers at the end of pregnancy; evaluating the change from baseline could help to clarify some of the reported associations. However, there is controversy about the longitudinal changes of these markers during pregnancy, where some authors describe increments towards the end [81,82], but others report no change [83,84]. Finally, the women in our study may not be representative of all women, since they were selected in a referral hospital, and may have higher risk factors for adverse perinatal outcomes.

One of the strengths of this study is the collection of the different variables with less bias since it is part of a prospective cohort. We were able to include different relevant sociodemographic, clinical, perinatal, and lifestyle confounding variables in the statistical models. The valid estimation of the usual intake is vital for studying the relationships between diet and health outcomes, where more diet assessments are required for greater precision [79]. Our data were obtained through the average of three diet recalls during pregnancy. In addition, multiple pass methodology was used, to reduce patient burden and to improve measurement accuracy [85]. Diet recalls were applied by well-trained nutrition professionals, in addition to the use of food replicas, standardized cups and spoons, and other methods to help to reduce bias in portion estimation. The 24 h recall is considered of “high potential” for collecting intake data when the objective is to analyze food processing [19]. Another strength was the method for selecting influential variables for the statistical models, which considered only the relevant variables through an automated data-driven process that is repeatable, replicable, exhaustive, and bias-free, instead of only manual selection.

## 5. Conclusions

UPF consumption represents around 30% of the total energy intake and improving the diet quality during pregnancy represents an area of opportunity for prevention. UPF consumption during pregnancy was inversely related to TAC, which may result in an increase in oxidative stress. Pregestational BMI and fiber were also associated with PC. Diet and maternal nutritional status are modifiable risk factors; it is important to optimize efforts in guaranteeing diet counselling, education, and dietary intervention in women before and during pregnancy. Limiting or avoiding UPF products during pregnancy may be a good target for nutrition intervention, which could promote a better antioxidant capacity in the body and limit OS damage and its associated consequences.

## Figures and Tables

**Table 1:** Consumption of UPFs according to maternal clinical and sociodemographic characteristics.

Factor	UPF Consumption (%TEI)		
	All (*n* = 119)	Lower Intake (<75°)	Higher Intake (≥75°)
Average UPF consumption	27.99 ± 10.72	23.22 ± 6.95	42.14 ± 6.59
Parity ^1^			
Nulliparous (*n* = 60)	27.01 ± 10.20	22.82 ± 7.37	40.72 ± 4.42
Multiparous (*n* = 52)	30.31 ± 10.92	24.49 ± 5.80	43.38 ± 7.97
Socioeconomic status ^1^			
Very low (*n* = 17)	27.40 ± 12.31	21.32 ± 7.80	41.97 ± 7.90
Low (*n* = 57)	28.36 ± 9.90	24.03 ± 6.49	41.62 ± 5.87
Middle–High (*n* = 44)	27.95 ± 11.73	22.97 ± 7.34	42.88 ± 7.42
Educational level ^1^			
Basic (*n* = 24)	27.23 ± 11.27	20.28 ± 4.92	41.12 ± 5.84
Middle (*n* = 45)	28.88 ± 12.33	23.00 ± 7.29	45.01 ± 8.12
Higher (*n* = 50)	27.57 ± 8.90	24.57 ± 7.11	39.51 ± 3.64
Occupation ^1^			
Housewife (*n* = 71)	26.93 ± 10.65	22.63 ± 6.54	43.02 ± 6.88
Student/Employee (*n* = 34)	29.27 ± 12.14	22.38 ± 8.09	41.89 ± 6.99
Pregestational BMI ^1^			
Normal (*n* = 50)	29.99 ± 10.69	24.72 ± 5.56	44.06 ± 7.54
Overweight (*n* = 43)	27.31 ± 10.51	22.84 ± 7.67	40.30 ± 5.62
Obesity (*n* = 26)	25.46 ± 10.86	21.04 ± 7.72	40.19 ± 4.92
GWG 3rd trimester ^1^			
Insufficient (*n* = 37)	27.53 ± 10.17	24.12 ± 5.50	39.86 ± 6.22
Adequate (*n* = 30)	27.20 ± 12.87	22.05 ± 7.22	44.98 ± 4.69
Excessive (*n* = 52)	28.78 ± 10.80	23.23 ± 7.82	42.45 ± 7.49
Maternal complications ^1^			
No (*n* = 94)	28.16 ± 10.52	23.49 ± 6.59	42.55 ± 6.51
Yes (*n* = 24)	27.99 ± 10.72	23.22 ± 6.95	42.14 ± 6.59
Multivitamin use (#trimesters) ^1^			
0–1 trimester (*n* = 39)	29.67 ± 11.23	24.41 ± 7.63	43.06 ± 6.89
2–3 trimesters (*n* = 80)	27.18 ± 10.43	22.68 ± 6.62	41.62 ± 6.54

Data presented as x ± SD. ^1^ Student’s *t*-Test or one-way ANOVA; not statistically significant (*p* > 0.05). UPF: ultra-processed food; %TEI: percentage of total energy intake; BMI: body mass index; GWG: gestational weight gain.

**Table 2:** Mean energy and macronutrient intake during pregnancy in all women and according to ultra-processed food intake.

Nutrient	UPF Consumption		
	All (*n* = 119)	Lower Intake (<75°)	Higher Intake (≥75°)
Energy (kcal)	2092.80 ± 544.54	2055.90 ± 480.41	2202.27 ± 699.71
Protein (g)	87.28 ± 25.09	86.85 ± 21.75	88.52 ± 33.52
Protein (%TEI)	16.98 ± 3.09	17.28 ± 3.19	16.06 ± 2.57
Carbohydrates (g)	270.36 ± 72.41	266.65 ± 67.62	281.35 ± 85.38
Carbohydrates (%TEI)	52.40 ± 6.73	52.32 ± 6.36	52.63 ± 7.82
Fiber (g)	25.41 ± 8.82	** 26.95 ± 8.36 **	20.85 ± 8.66 *^1^
Fat (g)	75.73 ± 26.18	73.65 ± 23.68	81.89 ± 32.15
Fat (%TEI)	31.86 ± 5.49	31.59 ± 5.28	32.15 ± 6.12
SFA (g)	21.35 ± 8.96	20.64 ± 7.92	23.43 ± 11.41
SFA (%TEI)	8.98 ± 2.30	8.89 ± 2.29	9.23 ± 2.32
MUFA (g)	24.39 ± 9.40	24.10 ± 8.77	25.23 ± 11.15
MUFA (%TEI)	10.19 ± 2.57	10.32 ± 2.56	9.79 ± 2.59
PUFA (g)	15.02 ± 6.39	14.49 ± 6.02	16.57 ± 7.25
PUFA (%TEI)	6.27 ± 2.12	6.15 ± 1.82	6.61 ± 2.84
TFA (g)	0.90 ± 0.93	0.83 ± 0.70	1.22 ± 1.38
TFA (%TEI)	0.39 ± 0.35	0.37 ± 0.33	0.45 ± 0.40
PUFA ω-3 (g)	1.38 ± 0.72	** 1.44 ± 0.73 **	1.16 ± 0 .65 *^2^
PUFA ω-3 (%TEI)	0.58 ± 0.28	** 0.62 ± 0.29 **	0.46 ± 0.18 *^2^
PUFA ω-6 (g)	8.06 ± 3.66	8.23 ± 3.85	7.56 ± 3.05
PUFA ω-6 (%TEI)	3.40 ± 1.08	** 3.51 ± 1.12 **	3.04 ± 0.84 *^1^
PUFA ω-6/ω-3 ratio	6.49 ± 2.54	** 6.23 ± 2.38 **	7.27 ± 2.88 *^2^

Data presented as x ± SD. ^1^ Student’s t-Test ^2^ Mann–Whitney U-test * *p* < 0.05. UPF: ultra-processed food; %TEI: total energy intake; SFA: saturated fatty acids; MUFA: monounsaturated fatty acids; PUFA: polyunsaturated fatty acids; TFA: trans fatty acids.

**Table 3:** Oxidative stress markers according to maternal clinical variables.

Characteristic	MDA (nmol/mg Dry Weight) (*n* = 119)		PC (nmol/mg Protein) (*n* = 119)		TAC (eq Trolox/mg Protein) (*n* = 119)	
All	0.444	(0.216)	8.943	(7.873)	0.070	(0.039)
UPF consumption						
Lower intake (<75°)	0.450	(0.231)	8.492	(6.856)	**0.070**	(0.039) *
Higher intake (≥75°)	0.403	(0.211)	13.289	(9.652)	**0.068**	**(0.021)**
Pregestational BMI						
Normal (*n* = 50)	**0.379**	(0.256) ^a^*	**7.400**	(6.173) ^c^**	0.070	(0.046)
Overweight (*n* = 43)	**0.416**	(0.218) ^b^*	**9.446**	(7.323) ^d^*	0.069	(0.033)
Obesity (*n* = 26)	**0.500**	**(0.140) ^ab^**	**14.528**	**(3.941) ^cd^**	0.070	(0.030)
GWG third trimester						
Insufficient (*n* = 37)	0.409	(0.264)	9.591	(7.869)	0.071	(0.036)
Adequate (*n* = 30)	0.460	(0.158)	8.092	(6.709)	0.073	(0.045)
Excessive (*n* = 52)	0.450	(0.217)	9.554	(8.530)	0.068	(0.030)
Maternal complications						
No (*n* = 94)	0.439	(0.220)	9.514	(7.725)	0.070	(0.039)
Yes (*n* = 24)	0.476	(0.194)	8.157	(9.643)	0.073	(0.050)
Multivitamin use (#trimesters)						
0–1 trimester (*n* = 39)	0.392	(0.246)	8.032	(7.957)	0.071	(0.042)
2–3 trimesters (*n* = 80)	0.467	(0.185)	9.554	(7.726)	0.070	(0.034)

Data presented as 50° (Interquartile range). Mann–Whitney U-test or Kruskall–Wallis test; * *p* < 0.05; ** *p* < 0.001. ^a–d^ Significant differences between the groups with the same letter. MDA: malondialdehyde; PC: protein carbonylation; TAC: total antioxidant capacity; UPF: ultra-processed food; BMI: body mass index; GWG: gestational weight gain.

**Table 4:** Association of UPF consumption with OS markers.

Predictive Variables	B	Standard Error	*p*	95% Confidence Interval		*R^2^*
				Lower Limit	Upper Limit	
UPF–MDA Model						0.227
Multivitamin use (# of trimesters; Ref: no use)						
1 trimesters	−0.0532	0.073	0.470	−0.199	0.092	
2 trimesters	0.039	0.118	0.743	−0.196	0.274	
3 trimesters	−0.0359	0.069	0.603	−0.172	0.101	
Pregestational BMI	0.0068	0.005	0.158	−0.003	0.016	
Fat (g)	0.0004	0.002	0.841	−0.004	0.005	
SFA (g)	0.0016	0.005	0.743	−0.008	0.011	
MUFA (g)	−0.0002	0.006	0.972	−0.013	0.013	
PUFA (g)	−0.0003	0.004	0.946	−0.009	0.009	
TFA (g)	0.0179	0.03	0.551	−0.041	0.077	
PUFA ω-3 (g)	0.0425	0.035	0.233	−0.028	0.113	
PUFA ω-6 (g)	−0.0102	0.006	0.110	−0.023	0.002	
Sleep score (3rd trimester)	0.0065	0.007	0.373	−0.008	0.021	
***UPF***	***−0.0052***	***0.001***	***<0.001***	***−0.007***	***−0.003***	
UPF–PC Model						0.243
Multivitamin use (# of trimesters; Ref: no use)						
1 trimester	−2.322	2.426	0.341	−7.134	2.489	
2 trimesters	2.1658	3.687	0.558	−5.145	9.477	
3 trimesters	−1.1352	2.374	0.634	−5.843	3.573	
***Pregestational BMI***	***0.4564***	***0.145***	***0.002***	***0.169***	***0.744***	
Fat (g)	0.0552	0.069	0.423	−0.081	0.191	
SFA (g)	0.0098	0.134	0.942	−0.255	0.275	
MUFA (g)	−0.1002	0.117	0.395	−0.333	0.132	
PUFA (g)	0.0347	0.149	0.816	−0.261	0.33	
Protein (g)	−0.0247	0.028	0.380	−0.08	0.031	
***Fiber (g)***	***0.1258***	***0.063***	***0.048***	***0.001***	***0.250***	
PUFA ω-3 (g)	−0.9474	0.751	0.210	−2.436	0.541	
PUFA ω-6 (g)	−0.2373	0.183	0.196	−0.599	0.125	
UPF	0.0061	0.041	0.883	−0.076	0.088	
UPF–TAC Model						0.109
Multivitamin use (# of trimesters; Ref: no use)						
***1 trimesters***	***0.016***	***0.007***	***0.017***	***0.003***	***0.029***	
2 trimesters	0.0153	0.01	0.133	−0.005	0.035	
***3 trimesters***	***0.0121***	***0.006***	***0.031***	***0.001***	***0.023***	
Age (years)	0.0009	0.001	0.153	0.000	0.002	
Pregestational BMI	−0.0002	0.000	0.679	−0.001	0.001	
Fat (g)	−0.0003	0.000	0.368	−0.001	0.000	
SFA (g)	0.0001	0.001	0.826	−0.001	0.001	
MUFA (g)	0.0004	0.001	0.565	−0.001	0.002	
PUFA (g)	0.0006	0.001	0.338	−0.001	0.002	
TFA (g)	0.001	0.003	0.725	−0.005	0.007	
PUFA ω-3 (g)	0.0034	0.003	0.321	−0.003	0.010	
PUFA ω-6 (g)	−0.0015	0.001	0.070	−0.003	0.000	
***UPF***	***−0.0005***	***0.000***	***0.002***	***−0.001***	***0.000***	

UPF: ultra-processed food; MDA: malondialdehyde; BMI: body mass index; SFA: saturated fatty acids; MUFA: monounsaturated fatty acids; PUFA: polyunsaturated fatty acids; TFA: trans fatty acids; CP: carbonylated proteins; TAC: total antioxidant capacity.

## Data Availability

The data presented in this study are available on request from the corresponding author. The data are not publicly available due to ethical reasons in accordance with consent provided by participants on the use of confidential data.
